# Robust half-metallicity and magnetic phase transition in Sr_2_CrReO_6_ via strain engineering

**DOI:** 10.1038/s41598-020-70768-7

**Published:** 2020-08-13

**Authors:** Qurat-Ul Ain, Shahnila Naseem, Safdar Nazir

**Affiliations:** Department of Physics, University of Sargodha, Sargodha, 40100 Pakistan

**Keywords:** Electronic structure, Ferromagnetism

## Abstract

Using ab-initio calculations, the electronic and magnetic properties of double perovskite oxide ${\text{Sr}}_2 {\text{CrReO}}_6$ with two type of strains: biaxial (along the [110]-direction) and hydrostatic (along [111]-direction) are investigated. The ground state of the unstrained system is half-metallic ferrimagnetic, due to a strong antiferromagnetic (AFM) coupling between Cr and Re atoms within both (GGA and GGA+*U*) exchange-correlation potentials. It is demonstrated that the robustness of half-metallicity can be preserved under the influence of both biaxial and hydrostatic strains. Interestingly, a transition from ferri-to-ferromagnetic is established due to Re spin flipping to that of the Cr ion (i.e. Cr and Re spin becomes parallel) within the GGA+*U* method for both biaxial and hydrostatic tensile strains of $\ge +2\%$. The strong confinement of orbitals due to tensile strain results in the decrease of electron hopping which further reduced the AFM coupling strength between Cr and Re atoms, this leads to a ferri-to-ferromagnetic transition. However, the GGA scheme holds the ferrimagnetic state with both kinds of strains. This work shows that tensile strain is a feasible way to optimize the magnetic properties of perovskite oxides, which are presumed to be beneficial for spintronic technology.

## Introduction

Half-metallic (HM) ferromagnetic (FM) materials (where one spin channel shows a metallic behavior and another one contains an insulating or semiconducting nature, refers to 100% spin polarized)^1–3^ have been attracted a great interest due to their functional technological applications in spintronics such as colossal magnetoresistance^4,5^ and spin-transfer torque magnetic random access memory^6–8^. As recently Kumari et al.^9^ experimentally observed that perovskite manganite family such as ${\text{La}}_{0.7} {\text{Sr}}_{0.3} {\text{MnO}}_3$ demonstrated the bipolar resistive switching behavior. Similarly, a recent review on two-dimensional spinterfaces and magnetic-interfaces exhibits a wide range of applications in spintronics such magnetic tunnel junctions^10^. In this respect, double perovskites ${\text{A}}_2 {\text{BB}}^{\prime} {\text{O}}_6$ (A = alkaline earth or rare earth metal atoms and ${\text{BB}}^{\prime} = 3/(4,5,6)\textit{d}$ transition metals such as $\text{B} = \text{Fe}, \text{Cr}, \text{Mn}, \text{Co}$, and Ni; ${\text{B}}^{\prime} = \text{Mo}, \text{Re}, \text{Os}$, and W) have been considered hot candidates because they show a HM ferrimagnetic (FiM) ground state having a relatively high Curie temperature ($T_c$) of 300–420 K^11–16^. In 1963, the discovery of metallic FiM with a reasonable $T_c$ of 410-450 K in ${\text{Sr}}_2 {\text{FeMoO}}_6$ (SFMO)^17,18^, substantially stimulated the researcher to synthesize or predict new double perovskites with novel magnetic properties.

Among the double perovskite oxides, Cr-based FiM ${\text{A}}_2 {\text{CrB}}^{\prime} {\text{O}}_6$ (A = Sr and Ca; ${\text{B}}^{\prime} = \text{Mo}$, W, and Re) exhibits potential applications in spintronic devices such as low-field magnetoresistive sensors or magnetic tunnel junctions^19^. Usually, ${\text{Cr}}^{+5}$ ($3d^5$ having $\text{S} = 5/2$) shows strong antiferromagnetic (AFM) coupling with ${\text{Mo}}^{+5}$ ($4d^5$ having $\text{S} = 1/2$), ${\text{W}}^{+5}$ ($5d^5$ having $\text{S} = 1/2$), and ${\text{Re}}^{+5}$ ($5d^5$ having $\text{S} = 1/2$) atoms, which results in FiM state. In this line, HM FiM or FM state having a total magnetic moment of 0.86(0.89) $\mu _B/\text{f.u}$ with a highest $T_C$ of 635 K is observed in ${\text{Sr}}_2 {\text{CrReO}}_6$ (SCRO)^12,20–22^ in contrast to very closely related ${\text{Ca}}_2 {\text{CrReO}}_6$, which shows insulating behavior in both experiments^12,20^ and theoretical work^23^. It is observed that SCRO contains very high $T_C$ as compared to other closely related ${\text{Sr}}_2 {\text{CrWO}}_6$ having a $T_C$ of 400 K^24,25^. However, Krockenberger et al.^26,27^ observed the highest $T_C$ of 725 K with high spin-polarization in ${\text{Sr}}_2 {\text{CrOsO}}_6$ and it is established that HM behavior in FiM double perovskite can be changed to insulating behavior. In contrast, the first-principles calculations exhibit the semi-metallic AFM state in the ${\text{Sr}}_2 {\text{Cr}} T {\text{O}}_6$ ($T = \text{Os}$ and Ru) system due to a strong spin-orbit coupling^28^. Moreover, a HM and metallic FiM (AFM coupling is favorable between Cr and Re atoms) states are predicted using density functional theory (DFT) calculations in SCRO with magnetic moments of 1.0 and $1.28 \, \mu _B$ with and without spin-orbit coupling, respectively^29^. Later on, Majewski et al. experimentally confirmed the FiM state in SCRO at a low temperature of 10 K^22^.

It is experimentally^30–37^ and theoretically^38–45^ found that strain often has a substantial influence on the electronic and magnetic properties of the systems, when an epitaxially films like ${\text{LaAlO}}_3$, SFMO, and ${\text{SrMoO}}_4$ grown on the ${\text{SrTiO}}_3$(MgO) substrate. As, Borges et al.^30^ experimentally observed that the magnetic moment in SFMO thin film reduces from its integral value due to strain exerted by substrates on film, which leads a transition from HM FM to FM metallic (FMM) state. Fix et al.^31^ experimentally and theoretically determined that HM state in SFMO thin film vanish, when it is grown on the ${\text{SrTiO}}_3$ (001) substrate. Moreover, the variation in the structural properties of SFMO thin film with biaxial strain is observed, when it is grown on different substrates such as MgO, ${\text{LaAlO}}_3$, and ${\text{SrTiO}}_3$^32^. It is theoretically found that a hydrostatic pressure upto 33-36 GPa on the SFMO, leads the Fe spin state from high to low and results in a transition from HM FiM to non-magnetic semiconductor state^39^. However, the system becomes metallic above 45 GPa. Similarly, Lu et al. theoretically predicted a magnetic phase transition in SFMO from FiM to FM for a tensile of $\ge + 8\%$, where Mo spin transform from intermediate-to-low spin state^41^. A recent experiment exhibits that SCRO film is showing the semiconducting behavior with energy gap ($E_g$) of 0.21 eV at a very low temperature of 2 K, when grown on the STO (lattice mismatch was only 0.05% between the film and the substrate)^46^. This is in contrast to the previous experiments^12,20,21,47,48^, which show HM FiM state. Along with this, a high pressure phase of SCRO has been studied both experimentally and theoretically by Olsen et al.^49^. It is found that system shows pseudocubic structure at low pressure, while the tetragonal phase becomes stable at high pressure. Hence, electronic and magnetic behvaior in these compounds is still under debate. Thus, the above mentioned experimental and theoretical facts motivated us to focus on the lesser-explored strained modulated physical properties of SCRO.

In this work, we studied the impact of two types of strains: biaxial and hydrostatic on the electronic and magnetic properties of SCRO employing first-principles DFT calculations. We proposed that by applying a biaxial tensile strain of $\ge + 2\%$, a magnetic transition from HM FiM to HMF state occurs due to Re spin transition from intermediate spin-state to low-spin state. On the other hand, a HM FiM to FMM transition is established for a hydrostatic tensile strain of $+ 2\%$, while for $\ge + 3\%$ system becomes HMF. This kind of spin flipping has many novel applications in data storage devices, switches, and optical displays^50^.

**Figure 1:**
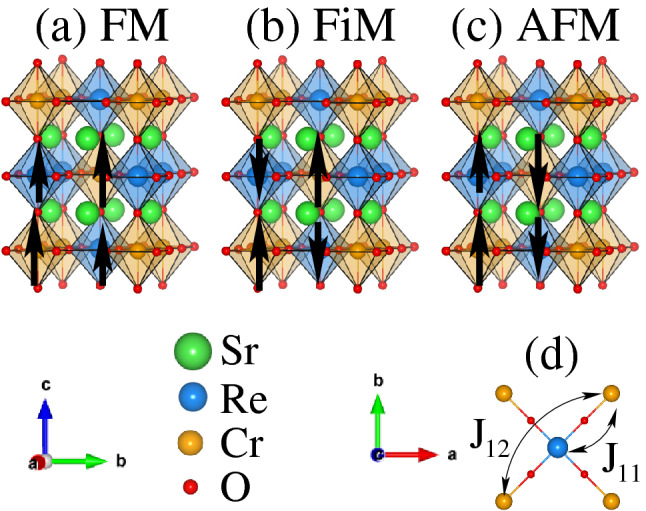
Schematic representation of the double perovskite structures of ${\text{Sr}}_2 {\text{CrReO}}_6$ in (**a**) ferromagnetic (FM), (**b**) ferrimagnetic (FiM), and (**c**) antiferromagnetic (AFM) ordering. The octahedral surroundings of the Cr and Re atoms are visualized in orange and blue colored polyhedron, respectively. (**d**) the top view in the *ab*-plane demonstrating the spin-coupling between Cr-Re (${\text{J}}_{11}$) via oxygen and indirect Cr-Cr (${\text{J}}_{12}$) atoms. The *x*, *y*, and *z*-axes are along the crystallographic *a*, *b*, and *c*-directions, respectively. The *x* and *z*-axis (*a* and *c*-directions) are pointing towards the observer in (**a**, **b**) and (**c**), respectively.

## Results and discussion

### Structural details

Bulk SCRO crystallizes in a tetragonal symmetry having space group No. 139 (I4/mmm) with experimental lattice constants of *a* = 5.52 and *c* = 7.82 Å^20^. The Sr, Cr, Re, O1, and O2 occupied 4*d*(1/2, 0, 1/4), 2*a*(0, 0, 0), 2*b*(0, 0, 1/2), 8*h*(0.249, 0.249, 1/2), and 4*e*(0, 0, 0.254) atomic positions, respectively. The schematic representation of SCRO crystal structures in FM, FiM, and AFM spin ordering are shown in Fig. 1a–c, respectively. For the case of FM structure, the spins of Cr and Re ions are align in-plane and out-of-plane directions and support with each other (see Fig. 1a). In FiM spin ordering, Cr and Re atoms are anti-align (see the arrowhead) within both in-plane and out-of-plane directions. However, Cr atoms are ferromagnetically (i.e. parallel to each other) coupled with each other at the diagonal. Similarly, Re atoms also have the same spin magnetization at the diagonal site, see Fig. 1b. In AFM spin ordering, Cr and Re atoms are anti-align and align for in-plane and out-of-plane directions, respectively. However, the diagonal atoms are showing the AFM coupling with each other, see Fig. 1c. It is very important to note that in both (FiM and AFM) magnetic ordering, the spin magnitude of Cr is larger than that of Re (compare the magnitude of the arrows in both cases). Along with this, two important couplings $J_{11}$ (between Cr and Re atoms via oxygen) $J_{12}$ (indirect coupling between Cr and Cr atoms) in the *ab*-plane also display in Fig. 1d.

### Unstrained system

**Figure 2:**
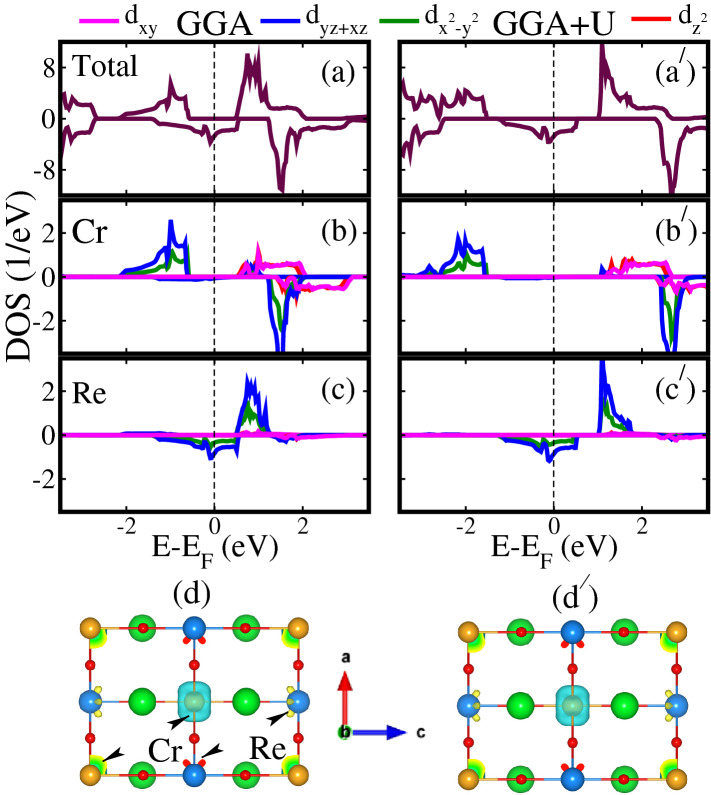
Calculated GGA/GGA+*U* spin-polarized (**a**, **a**$^{\prime}$) total and orbital resolved partial (**b**, **b**$^{\prime}$) Cr 3*d* and (**c**, **c**$^{\prime}$) Re 5*d* density of states (DOS) of ${\text{Sr}}_2 {\text{CrReO}}_6$ in a FiM spin oredering. (**d**, **d**$^{\prime}$) represents the spin density difference (difference between spin-majority and spin-minority channels) for the top view in the *ac*-plane. The same iso-value of $0.05\textit{e}/{\AA }^3$ is used to produce these spin distribution plots. The Fermi level is shown by the vertical dashed line at 0 eV in each DOS figure. The *x*, *y*, and *z*-axes are along the crystallographic *a*, *b*, and *c*-directions, respectively. The *y*-axis (*b*-direction) is pointing towards the observer.

First, we determined the energetically stable magnetic ordering in SCRO by comparing the total energies of FM, FiM, and AFM states as $\Delta E_1/\Delta E_2 = E_{FiM}-E_{FM}/E_{FiM}-E_{AFM}$. The computed values of $\Delta E_1/\Delta E_2$ are $-3.60/-3.72$ and $-3.35/ -4.41 \, \text{meV}$ having corresponding $T_{C1}$ and $T_{C2}$ 27.9/28.8 and 27.1/34.1 K within GGA and GGA+*U* methods, respectively. The $T_C$ is calculated by using the Eqn. 2 as mention in the recent work of Nair et al.^51^. The “−” sign of $\Delta E$ indicates that AFM coupling between diagonal Cr atoms is not favorable and also between Re (i.e. both Cr and Re spins wants to align in the same direction at the diagonal). Moreover, this also shows that Cr and Re spins want to be remain anti-align along the in-plane as well as out-of-plane directions. Therefore, FiM magnetic spin ordering is energetically more stable as compared to FM and AFM structures in both cases. Hence, FiM magnetic structure of SCRO is taken into account for further investigations. The calculated spin-polarized total and orbital resolved partial density of states (TDOS and PDOS) of unstrained (0%) SCRO within stable FiM spin ordering for GGA and GGA+*U* methods are shown in Fig. 2. From Fig. 2a, ${\text{a}}^{\prime}$, one can see that the system exhibits HM behavior within both GGA and GGA+*U* methods, respectively. Spin minority TDOS are contributing to the metallicity, while majority channel remains insulator in both cases. However, the computed $E_g$ of 2.59 eV in a spin-majority channel with GGA+*U* is much higher than that of 1.01 eV for GGA. Our calculated TDOS are in good agreement with the previously reported theoretical DFT works^29,52–54^ and experimental observations^12,21,22^, where the authors confirm the HM nature of the system.

**Figure 3:**
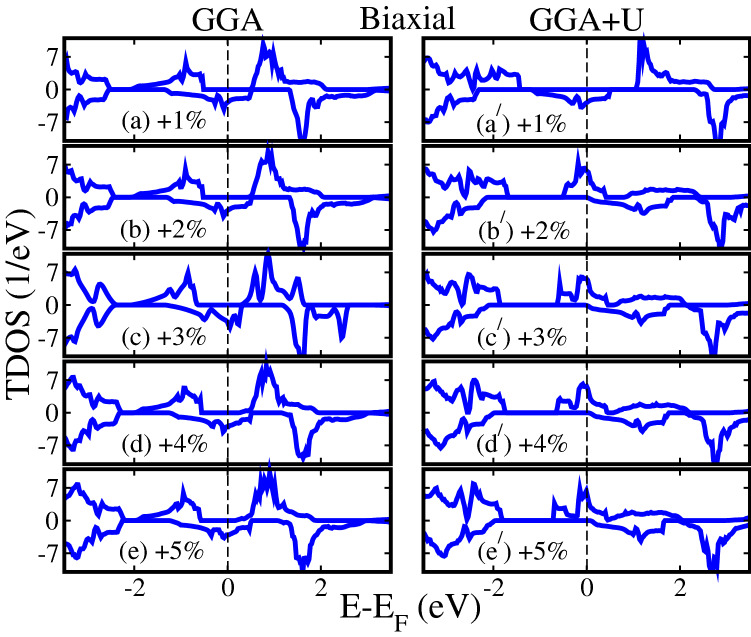
Calculated GGA/GGA+*U* spin-polarized total density of states (TDOS) of ${\text{Sr}}_2 {\text{CrReO}}_6$ for (**a**, **a**$^{\prime}$) $+1\%$, (**b**, **b**$^{\prime}$) $+2\%$, (**c**, **c**$^{\prime}$) $+3\%$, (**d**, **d**$^{\prime}$) $+4\%$, and (**e**, **e**$^{\prime}$) $+5\%$ biaxial tensile strains along [110]-direction (in the *ab*-plane).

In a perfect octahedral crystal field, the Cr/Re 3/5*d* states split into triply $t_{2g}$ ($d_{xy}$, $d_{xz}$, and $d_{yz}$) and doubly $e_g$ ($d_{x^2-y^2}$ and $d_{3z^2-r^2}$) degenerate states. However, here ${\text{CrO}}_6/{\text{ReO}}_6$ octahedron distorted and ${\text{O}}_h$ symmetry reduced to ${\text{D}}_4h$, which degraded the Cr/Re 3/5*d* orbitals degeneracy. Therefore, the $t_{2g}$ states further split into singlet $d_{xy}$ and doubly ($d_{xz}$ and $d_{yz}$) non-degenerate states. Similarly, $e_g$ states split into singlet $d_{x^2-y^2}$ and $d_{3z^2-r^2}$ non-degenerate states. Our results show that Cr 3*d* orbitals are not contributing to the metallic channel (spin-minority) within both GGA and GGA+*U* as display in Fig. 2b, ${\text{b}}^{\prime}$, respectively. On the other hand, Re $d_{yz+xz}$ and $d_{x^2-y^2}$ are mainly responsible for conductivity in the spin-minority channel and also show a strong hybridization around the Fermi level ($E_F$) for both GGA and GGA+*U* methods as shown in Fig. 2c, ${\text{c}}^{\prime}$, respectively. Moreover, Re $d_{yz+xz}$ and $d_{x^2-y^2}$ states become more confine near $E_F$ in the case of GGA+*U* as compared to GGA, which enhanced the magnitude of the moment. For more deep understanding, we plotted the spin difference densities (the difference between spin-majority and spin-minority channels) plots for the top view in the *ac*-plane within GGA and GGA+*U* approaches in Fig. 2d, ${\text{d}}^{\prime}$, respectively. The more densities around Cr atoms in both cases confirm that main contributions to the total magnetic moment comes from Cr with a small from Re ion, whcich confirms the DOS in Fig. 2b, b$^{\prime}$, c, c$^{\prime}$.

The calculated total magnetic moment is $1.0 \, \mu _B/\text{f.u}$. within both GGA and GGA+*U* methods, which confirms the HM nature of SCRO and also in a good agreement with experimentally/theoretically found value of $0.9/1.0 \, \mu _B/\text{f.u}$.^21,48^. The partial moments on the Cr and Re atoms are 2.09/2.44 and $-0.83/ -1.06 \, \mu _B$ for GGA/GGA+*U* scheme, respectively. The “−” sign in Re moment indicates that its spin is ant-align as well as with smaller in magnitude than that of Cr, which confirms the FiM ordering in SCRO. Our computed Cr(Re) moments are very close to the previously calculated^29,52–54^ and experimentally^22,48^ observed values.

Next, we investigated the influence of biaxial (along the [110]-direction) and hydrostatic strains (along the [111]-direction) on the electronic and magnetic properties of SCRO. We modeled the biaxial and hydrostatic strains in the range of $-5\%$ to $+5\%$ by tunning the lattice parameters in the *ab*-plane and along the three axis (*x*, *y*, *z*), respectively. The “−” and “$+$” signs indicate compressive and tensile strains, respectively.

**Figure 4:**
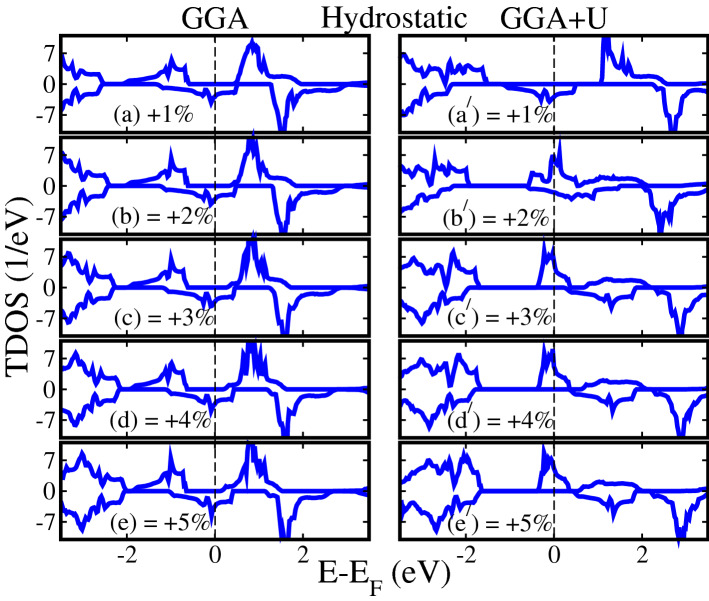
Calculated GGA/GGA+*U* spin-polarized total density of states (TDOS) of ${\text{Sr}}_2 {\text{CrReO}}_6$ for (**a**, **a**$^{\prime}$) $+1\%$, (**b**, **b**$^{\prime}$) $+2\%$, (**c**, **c**$^{\prime}$) $+3\%$, (**d**, **d**$^{\prime}$) $+4\%$, and (**e**, **e**$^{\prime}$) $+5\%$ hydrostatic tensile strains along [111]-direction.

**Figure 5:**
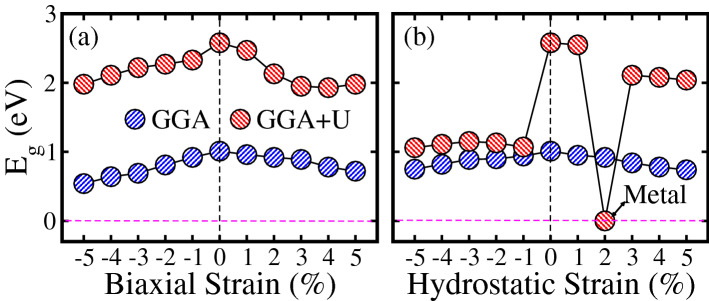
Calculated GGA and GGA+*U* energy band gap ($E_g$) in the spin-majority/minority channel of ${\text{Sr}}_2 {\text{CrReO}}_6$ as a function of (**a**) biaxial [110] and (**b**) hydrostatic [111] strains.

**Figure 6:**
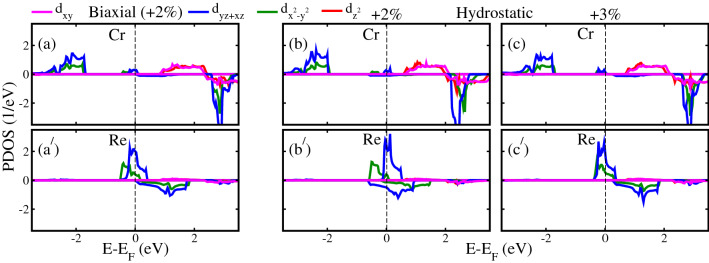
Calculated GGA+*U* spin-polarized orbital resolved partial density of states (PDOS) of Cr/Re 3/5*d* in ${\text{Sr}}_2 {\text{CrReO}}_6$ for (**a**, **a**$^{\prime}$) biaxial $+2\%$ and hydrostatics (**b**, **b**$^{\prime}$) $+2\%$ and (**c**, **c**$^{\prime}$) $+3\%$ tensile strains.

### Strained systems

#### Structure stability

First, we examined the structural stability of the unstrained and strained SCRO systems in a FiM spin ordering by calculating the enthalpies of formation ($\Delta H_f$) as:

1 $$\begin{aligned} \Delta H_f = E_t^{Sr_2CrReO_6} - \frac{1}{2}E_t^{Sr} - E_t^{Cr} - E_t^{Re} - \frac{6}{2}E_t^{O_2} \end{aligned}$$

where $E_t^{Sr_2CrReO_6}$ is the total energy of SCRO, while $E_t^{Sr}$, $E_t^{Cr}$, $E_t^{Re}$, and $E_t^{O_2}$ are the per-atom energies of the most stable low-temperature phases of Sr, Cr, Re, and O ions, respectively. The calculated $\Delta H_f$ of SCRO as a function of biaxial and hydrostatic strains within both (GGA and GGA+*U*) schemes are plotted in the Supporting Information (SI) of Fig. 1Sa, b, respectively. One can see that the negative values of $\Delta H_f$ in all cases confirmed the structure stability of the systems with respect to applied strains and can be easily grow in the experiment. However, biaxial strain systems show more stability because of lower energies as compared to hydrostatic ones (correlate the Fig. 1Sa, b in the SI). Our results also show that structure stability increases when both biaxial and hydrostatic strains are adjusted from $-5\%$ to $+5\%$, as $\Delta H_f$ becomes more negative. Furthermore, it is also noted that GGA+*U* method produces lower $\Delta H_f$ values than that of GGA in both types of strains (biaxial and hydrostatic), which means that systems exhibit higher solidity in this scheme.

### Electronic properties

To qualitatively shows the changes in the electronic properties of SCRO with various biaxial strains, we produced the spin-polarized TDOS for biaxial tensile strains of $+1\%$, $+2\%$, $+3\%$, $+4\%$, and $+5\%$ in Fig. 3a, a$^{\prime}$, b, b$^{\prime}$), c, c$^{\prime}$, d, d$^{\prime}$, and e, e$^{\prime}$) within GGA/GGA+*U* method, respectively. The GGA calculated TDOS for biaxial tensile strains of $+1\%$ to $+5\%$, almost showing the same behavior as found in unstrained one (0%), compare Fig. 3a–e with Fig. 2a. A similar pattern of TDOS computed with GGA for biaxial compressive strains (ranging from $-1\%$ to $-5\%$) is also found, see Fig. 2S of SI. This means that GGA keeps the HM nature of SCRO as a function of both (compressive and tensile) biaxial strains (Fig. 2Sa, b and Fig. 3a–e) as determined in unstrained system (Fig. 2a), where the spin-majority channel is insulator and spin-minority is metallic. In the case of GGA+*U* method, biaxial tensile strain of $+1\%$ produces almost the same TDOS as found in unstrained (0%) system (compare Figs. 3${\text{a}}^{\prime}$ and 2${\text{a}}^{\prime}$). Surprisingly, the spin-majority channel becomes metallic and spin-minority is showing the insulating behavior with a large $E_g$ of 2.14 eV for a biaxial tensile strain of $+2\%$ to $+5\%$ (see Fig. 3b$^{\prime}$–e$^{\prime}$)) in contrast to calculated GGA TDOS for $-1\%$ to $-5\%$ (Fig. 2Sa–e of the SI) and $+1\%$ to $+5\%$ (Fig. 3a, e) as well as with GGA+*U* scheme for $-1\%$ to $-5\%$ (Fig. 2S${\text{a}}^{\prime}$–${\text{e}}^{\prime}$ of the SI) and $+1\%$ (Fig. 3${\text{a}}^{\prime}$) biaxial strains. Hence, one can conclude that the system maintains its HM nature by adjusting the biaxial strain of $-5\%$ to $+5\%$ along the [110]-direction (in the *ab*-plane) within both methods.

Next, the calculated spin-polarized TDOS for hydrostatic tensile strains of $+1\%$, $+2\%$, $+3\%$, $+4\%$, and $+5\%$ are plotted in Fig. 4a, a$^{\prime}$, b, b$^{\prime}$, c, c$^{\prime}$, d, d$^{\prime}$, e, e$^{\prime}$ within GGA/GGA+*U* method, respectively. Similar to biaxial strain results, the TDOS produced with GGA for the hydrostatic strain of $+1\%$ to $+5\%$ (see Fig. 4a–e), almost showing the identical pattern as found in the unstrained (0%) system (Fig. 2a). Moreover, calculated GGA+*U* calculated TDOS for the hydrostatic strain of $+1\%$ also exhibits the same trend (compare Figs. 4${\text{a}}^{\prime}$ and 2${\text{a}}^{\prime}$). Interestingly, a HM to metallic and then metallic to HM transition is established for the hydrostatic tensile strains of $+2\%$ and $+3\%$ to $+5\%$ for GGA+*U* method as plotted in Fig. 4${\text{b}}^{\prime}$ and ${\text{c}}^{\prime}$ to ${\text{e}}^{\prime}$, respectively. At $+2\%$ hydrostatic tensile strain, spin-majority channel becomes metallic as found in the case of $+2\%$ biaxial tensile strain for GGA+*U* (Fig. 3${\text{b}}^{\prime}$). However, few spin-minority TDOS are also crossing the $E_F$ from conduction to valence band (see Fig. 4${\text{b}}^{\prime}$), which makes the system metallic in contrast to biaxial strain (Fig. 3${\text{b}}^{\prime}$). Surprisingly, the spin-minority TDOS shifts toward the higher energies in the conduction band for $+3\%$ hydrostatic tensile strain with $E_g$ of 2.12 eV and system becomes again HM (Fig. 4${\text{c}}^{\prime}$) and also for higher strains (i.e. $+4\%$ (Fig. 4${\text{d}}^{\prime}$) and $+5\%$ (Fig. 4${\text{e}}^{\prime}$). Moreover, for hydrostatic compressively strain systems ranging from $-1\%$ to $-5\%$, the GGA and GGA+*U* calculated spin-polarized TDOS are plotted in Fig. 3Sa–e and ${\text{a}}^{\prime}$–${\text{e}}^{\prime}$ of the SI, respectively, which almost showing the same behavior as found in the case of biaxially strained systems (Fig. 2S of the SI).

**Figure 7:**
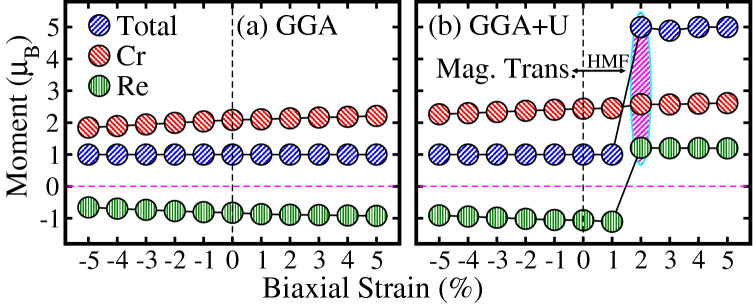
Calculated total and partial magnetic moments of ${\text{Sr}}_2 {\text{CrReO}}_6$ within (**a**) GGA and (**b**) GGA+*U* schemes as a function of biaxial strains along the [110]-direction. The “−” and “$+$” signs indicate compressive and tensile strains, respectively. The shaded region (magenta color in **b**) represents the magnetic transition from half-metallic ferri-(HM FiM) to half-metallic ferromagnetic (HMF) state.

The computed $E_g$ in the spin-majority or spin-minority channel within GGA/GGA+*U* method for biaxial and hydrostatic strained systems are plotted in Fig. 5a,b, respectively. In the case of biaxial strain, a smooth decrease in $E_g$ is evident for both (compressive and tensile) strains within GGA/GGA+*U* scheme, see Fig. 5a. A similar trend of reduction in $E_g$ as a function of both (compressive and tensile) hydrostatic strain systems is also predicted (see Fig. 5b). However, a sharp decrease in $E_g$ is established for a compressive strain of $-1\%$ within GGA+*U* in contrast to biaxially compressive strained system and then a very small decline in $E_g$ is embedded upto $-5\%$. It is important to note that $E_g$ becomes zero for $+2\%$ hydrostatic tensile strained system (Fig. 5b), which means that the system exhibits FM metallic behavior as shown in TDOS of Fig. 4${\text{b}}^{\prime}$.

The decrease in the $E_g$ can be explained by understanding the mutual hybridization between Cr/Re 3/5*d* and O 2*p* states. For this, we produced Cr 3*d*, Re 5*d*, and O 2*p* partial density of states (PDOS) for unstrained (0%) and biaxially compressive and tensile strained systems for instant in Figs. 4S and 5S of the SI within GGA scheme, respectively. From Fig. 4S, one can clearly see that partially occupied Cr 3*d* orbitals in the valence band strongly shift towards the higher energies as compared to unstrained (0%) system, when a biaxial compressive strain is adjusted from $-1\%$ to $-5\%$ (Fig. 4Sb–f of the SI), substantially reduced the $E_g$. This is attributed to a strong hybridization between Cr 3*d* and O 2*p* states because compressive strain enhanced the interaction between atoms and more orbitals overlap with each other. This results in the broadening of the bandwidth and decrease in $E_g$ as discussed in these Refs.^41,55^. Moreover, a small shift of partially unoccupied Re 5*d* bands towards the lower energies in the conduction band can be seen, which also contribute to the reduction of $E_g$. Black dotted lines on the band edges of Cr and Re atoms in the valence and conduction bands are drawn for a guide to the eye, which clearly shows the shift of bands towards higher and lower energies, respectively. In the case of a tensile strain of $+1\%$ to $+5\%$, Cr and Re bands position in the valence and conduction bands almost remains the same (Fig. 5Sb–f of the SI) as that of the unstrained system (Fig. 5Sa of the SI). However, a small shift of partially unoccupied Cr 3*d* orbitals in the conduction band from higher to lower energies occurs, which leads to a minute reduction of $E_g$ (Fig. 5a). Hence, the decrease in $E_g$ as a function of both type of strains in the remaining systems can also be explained as above discussed for a biaxially strained system within GGA approach.

**Figure 8:**
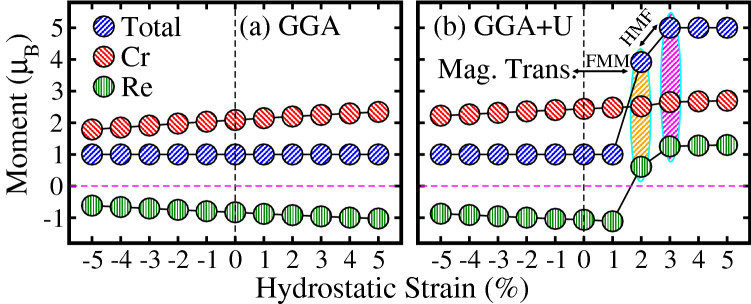
Calculated total and partial magnetic moments of ${\text{Sr}}_2 {\text{CrReO}}_6$ within (**a**) GGA and (**b**) GGA+*U* schemes as a function of hydrostatic strains along the [111]-direction. First shaded region (orange color in **b**) represents the magnetic transition from half-metallic ferri- (HM FiM) to ferromagnetic metallic (FMM) state, while second (magenta color) indicates the FMM to HM ferromagnetic (HMF) transition.

To deeply understand the origin of the flipping of metallic electronic states from spin-minority to spin-majority channel in TDOS (Figs. 3${\text{b}}^{\prime}$, 4${\text{b}}^{\prime}$, ${\text{c}}^{\prime}$) at the critical biaxial ($+2\%$) and hydrostatic ($+2\%$ and $+3\%$) tensile strains within GGA+*U* method, we produced the orbital resolved partial Cr 3*d* and Re 5*d* PDOS in Fig. 6. In the case of $+2\%$ biaxial strain system, a small contribution to the metallicity in the spin-majority channel comes from Cr $d_{yz+xz}$ orbitals (Fig. 6a), while Re $d_{x^2-y^2}$ and $d_{yz+xz}$ orbitals are mainly responsible for metallicity and also showing a strong hybridization near $E_F$ (Fig. 6${\text{a}}^{\prime}$). It is important to note that Cr and Re states are not crossing $E_F$ in the spin-minority channel, therefore this system is referred to HM. Moreover, Re $d_{x^2-y^2}$ and $d_{yz+xz}$ metallic states flip from spin-minority to the spin-majority channel at a critical biaxial strain of $+2\%$ and also for higher strains as compared to unstrained (Fig. 2${\text{c}}^{\prime}$) and compressive/tensile strained system (Fig. 3). For the hydrostatic $+2\%$ tensile strain, a very small contribution to metallicity comes from Cr $d_{yz+xz}$ orbitals in the spin-majority channel (Fig. 6b) as found in the case of $+2\%$ biaxial strain system (Fig. 6a). However, Re $d_{x^2-y^2}$ and $d_{yz+xz}$ orbitals are mainly responsible for metallicity in the spin-majority channel along with a substantial contribution from spin-minority $d_{yz+xz}$ orbitals (Fig. 6${\text{b}}^{\prime}$) in contrast to biaxial $+2\%$ tensile strain (Fig. 6${\text{a}}^{\prime}$). As both channels are metallic, therefore, this system is referred to FMM for $+2\%$ hydrostatic tensile strain. Similarly, almost a negligible contribution to metallicity comes from Cr spin-majority $d_{yz+xz}$ states for $+3\%$ hydrostatic tensile strain (see Fig. 6c), while the spin-minority channel is insulator. On the other hand, Re $d_{x^2-y^2}$ and $d_{yz+xz}$ orbitals in the spin-majority channel are primarily responsible for the conductivity as shown in Fig. 6${\text{c}}^{\prime}$. Interestingly, the spin-minority channel becomes insulator (no states are crossing the $E_F$) again for $+3\%$ tensile strain, which leads the system from HM to FM state.

### Magnetic properties

Next, we examined the strain-induced changes of the magnetic moments in the SCRO system. The computed total and partial magnetic moments on the Cr/Re ions as a function of biaxial strains are plotted in Fig. 7. In the case of GGA, one can clearly see that Cr and Re magnetic moments are slight decreases from 2.09 to $1.85 \, \mu _B$ and $-0.83$ to $-0.66 \, \mu _B$ when a biaxial strain is adjusted from 0% to $-1.5\%$ (Fig. 7a), respectively. On the other hand, moments slightly increases from 2.09 to $2.22 \, \mu _B$ and $-0.83$ to $-0.93 \, \mu _B$ in the range of 0% to $+ 1.5\%$ (Fig. 7a), respectively. The “−” sing in the Re moments indicate that Cr and Re spins are anti-align (anti-parallel) and AFM coupling between them is favorable, which confirms the FiM magnetic ordering under-considered both compressive and tensile biaxial strains within GGA scheme as found in the case of the unstrained system. Similarly, a slight decrease (increase) in the magnitude of Cr and Re moment is evident for GGA+*U* method when a biaxial strain is adjusted from 0% to $-5\%$ (0% to $+5\%$), see Fig. 7b. Here, it is very important to note that Re moment changes its sign from “−” to “$+$” at a critical $+2\%$ tensile strain, which means that Re spin is parallel to that of the Cr ion and this leads the magnetic phase transition from FiM to FM ordering. A similar trend is also established for $+3\%$ to $+5\%$ biaxial tensile strained systems. However, the total moment remains constant to an integral value of $1.0 \, \mu _B$ for both GGA and GGA+*U* methods for strain ranges of $-5\%$ to $+5\%$ and $-5\%$ to $+1\%$, respectively. In contrast, a total moment increases to an integral value of $5.0 \, \mu _B$ within GGA+*U* scheme for the biaxial tensile strains of $+2\%$ to $+5\%$. Because Re moment changes its sign from negative to positive and added to that of the Cr, which provides strengthen to the total moment. These integral values of the moments in all strained systems, confirms the HM nature as display in Fig. 3. Similarly, Lu et al.^41^ also established a magnetic transition from FiM to FM in the SFMO system for a biaxial tensile strain of $+ 7\%$ using DFT calculations. As one can see that in the SFMO system, transition occurs at a very high biaxial tensile strain of $+ 7\%$. However, our calculations reveal that a moderate biaxial strain of $+2\%$ is high enough to make a transition from HM FiM to HM FM in SCRO system, which is practically more feasible as found in the case of SFMO system.

**Figure 9:**
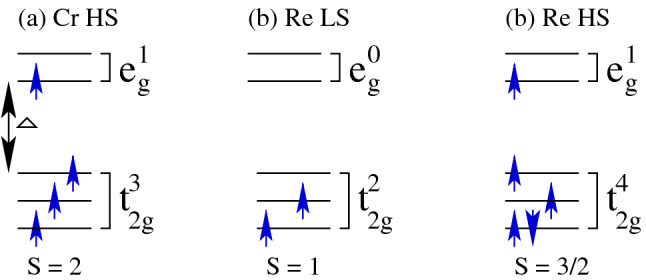
Schematic representation of (**a**) Cr high spin (HS), (**b**) Re low spin (LS), and Re intermediate spin (IS) states. $\Delta$ represents the enegy gap between $t_{2g}$ and $e_g$ states.

Similary, the GGA calculated magnetic moments for both (compressive and tensile) hydrostatic strains exhibit a comparable trend and magnitudes (Fig. 8a) as found in the case of biaxial strain systems (Fig. 7a), as discussed above. The moments (total and partials) increases (decreases), when strain is adjusted from $-5\%$ to 0% (0% to $+5\%$). For GGA+*U* scheme, the computed moments for hydrostatic strains of $-5\%$ to $+1\%$ (Fig. 8b) show the same behavior as established in the case of biaxially strained systems (Fig. 7b). Surprisingly, Re moment changes its sign from negative to positive at a critical tensile strain of $+2\%$, which means that Re spin is parallel to that of Cr and results in a magnetic transition from FiM to FM. However, the total moment is $3.92 \, \mu _B$ at this strain, which is not an integral value and also in contrast to biaxial $+2\%$ tensile strain system. Therefore, it is referred to FMM instead of HMF and confirms the calculated TDOS in Fig. 4${\text{b}}^{\prime}$. Interestingly, the system displays a second magnetic transition from FMM to HFM ordering at a tensile strain of $+3\%$ with a total integral moment of 5.0 $\mu _B$. Because, Re moment sharply increases from 0.62 to 1.25 $\mu _B$ when strain is adjusted from $+2\%$ to $+3\%$, which gives strengthen to the total moment (Fig. 8b). Hence, this integral moment leads the system to HMF ordering as displayed in TDOS of Fig. 4${\text{c}}^{\prime}$. Moreover, a similar pattern of moments is also established for hydrostatic tensile strains of $+4\%$ and $+5\%$ within GGA+*U* method, see Fig. 8b.

From the above calculated average moments of Cr (${\sim } 2.0/2.4 \, \mu _B$) and Re (${\sim } 0.9/1.1 \, \mu _B$) in both types of strains as well as in unstrained system within GGA/GGA+*U*, except for hydrostatic tensile strain of $+2\%$ in which Re moment is $0.61 \, \mu _B$, which means that Cr and Re atoms remain in the high and low spin (HS and LS) states, respectively. However, Re is almost in low spin (LS) state only for hydrostatic tensile strain of $+2\%$. Usually, Cr HS, Re LS, and Re HS are described as $3d^4$ ($t_{2g}^3 \, e_g^1$) with $\text{S} = 2$, $5d^5$ ($t_{2g}^2 \, e_g^0$) with $\text{S} = 1$, and $5d^5$ ($t_{2g}^4 \, e_g^1$) with $\text{S} = 3/2$ spin states as shown in the schematic diagram of Fig. 9a–c, respectively.

**Figure 10:**
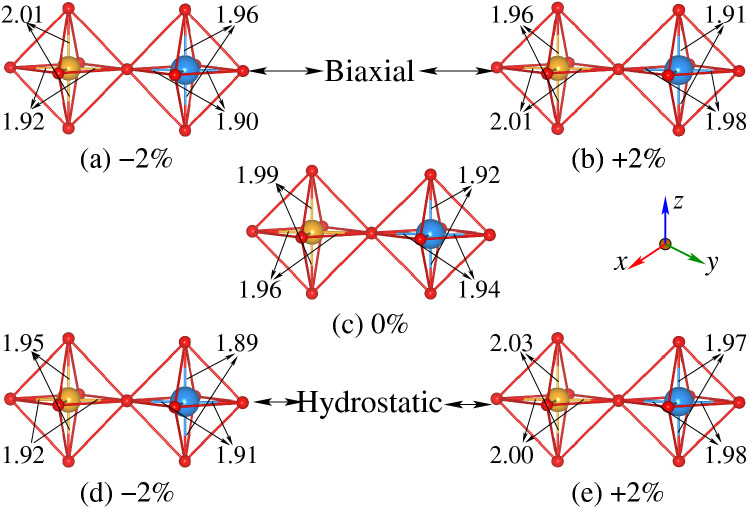
Locally distorted ${\text{CrO}}_6 ({\text{ReO}}_6)$ octahedron in ${\text{Sr}}_2 {\text{CrReO}}_6$ systems for (**a**) $-2\%$ and (**b**) $+2\%$ biaxial strains along the [110]-direction, (**c**) 0% (unstrained), and (**d**) $-2\%$ and (**e**) $+2\%$ hydrostatic strains along the [111]-direction. The *x*, *y*, and *z*-axes are along the crystallographic *a*, *b*, and *c*-directions, respectively.

### Octahedra distortion

The moment variations with strain can be understood from the structural distortion of the ${\text{CrO}}_6 ({\text{ReO}}_6)$ octahedron. Therefore, we plotted the locally distorted ${\text{CrO}}_6 ({\text{ReO}}_6)$ octahedron for different strained systems in Fig. 10. The ${\text{CrO}}_6 ({\text{ReO}}_6)$ octahedra of the biaxially compressive ($-2\%$) and tensile ($+2\%$) strained systems are displayed in Fig. 10a,b, while those of the hydrostatic compressive ($-2\%$) and tensile ($+2\%$) strained systems are depicted in Fig. 10d, e, respectively. For comparison, the distorted ${\text{CrO}}_6 ({\text{ReO}}_6)$ octahedron in the unstrained (0%) system is shown in Fig. 10c. Our results show that Cr–O/Re–O bond length substantially increases from 1.92/1.90 Å to 1.96/1.94 Å in the *ab*-plane when driving from biaxially compressive ($-2\%$) to tensile ($+2\%$) strained systems (see Fig. 10a,b). Due to the expansion/reduction of Cr–O and Re–O bond lengths in the tensile/compressive ($+2\%/-2\%$) strained system (Fig. 10a, b) as compared to unstrained (0%) one (Fig. 10c), results in decreased/increased of the electrons hoping. Therefore, Cr and Re moments becomes larger and smaller due to highly confined and unconfined of spatial charge distribution around these atoms, respectively. Moreover, small elongation and suppression of Cr–O/Re–O bond length in the *c*-axis for biaxial compressive (Fig. 10a) and tensile (Fig. 10b) is evident than that of the unstrained system (Fig. 10c), respectively.

In the case of compressive ($-2\%$) hydrostatic strain, Cr–O/Re–O bond length shorten along the *ab*-plane (Fig. 10d) as compared to the unstrained system (Fig. 10c), which is almost similar to the biaxially compressive ($-2\%$) strained system (Fig. 10a). Along with this, Cr–O/Re–O bond length also decreases along the *c*-axis for $-2\%$ strain (Fig. 10d) as compared to unstrained one (Fig. 10c), which is in contrast to biaxially compressive strained system (Fig. 10a). The decrease in the bond lengths along the *ab*-plane results in smaller Cr/Re moments due to degradation of the spatial charge distribution as discussed above. However, Cr/Re moments increases because of the lengthening of the Cr–O/Re–O bond length along the [110]-direction for hydrostatic tensile ($+2\%$) strain (Fig. 10e) than that of the unstrained system (Fig. 10c), as also determined in the case of biaxially tensile ($+2\%$) system (Fig. 10b). Furthermore, the Cr–O/Re–O bond length also increases along the *c*-axis for hydrostatic tensile ($+2\%$) strain, which is in contrast to biaxial tensile ($+2\%$) strain system. Hence, one can conclude that elongation and contraction of bond lengths can substantially be enhanced and reduced the magnetic moments due to confinement and degradation of spatial charge distribution, respectively.

## Conclusion

In summary, the electronic and magnetic properties of unstrained and strained (biaxial and hydrostatic) ${\text{Sr}}_2 {\text{CrReO}}_6$ systems were investigated using spin-polarized density functional theory. Our calculations revealed that antiferromagnetic (AFM) coupling between Cr and Re, while ferromagnetic (FM) couplings are energetically favorable between the similar (Cr/Cr and Re/Re) ions, results in ferrimagnetic (FiM) ground state for the unstrained SCRO system. Furthermore, it is demonstrated that only Re spin-minority $d_{yz+xz}$ and $d_{x^2-y^2}$ states are mainly responsible for metallicity, which leads the system to half-metal (HM) FiM. The HM nature shows the robustness as a function of both kinds of strains. Interestingly. a magnetic transition from HM FiM to HM FM is established for $+2\%$ biaxial tensile strain within GGA+*U* method due to flipping of Re spin alignment to that of Cr ion. On the other hand, two magnetic transitions: (1) a HM FiM to FM metal (FMM) and (2) FMM to HM FM are found in the case of hydrostatic tensile strains of $+2\%$ and $+3\%$ for GGA+*U* method, respectively. Strong confinement of orbitals due to tensile strains, leads to the decrease of electron hoping and also reduced the AFM coupling strength between Cr and Re atoms, which results in FiM-to-FM transition. Thus, we propose that magnetic properties of the perovskite oxide systems can be optimized by applying a moderate tensile strain along the [110] and [111]-directions.

## Computational methods

Full-potential linearized augmented plane wave method of spin-polarized DFT as implemented in the WIEN2K code^56^ is used. The generalized gradient approximation (GGA), which is parameterized by Perdew-Burke-Ernzerhof (PBE)^57^ plus on-site Coulomb interaction (GGA+*U*) approach was employed with *U* = 3.5 and 2.4 eV for Cr 3*d* and Re 5*d* states, respectively^58^. For the wave function expansion inside the atomic spheres, a maximum value of $l_{max} = 12$ is chosen and the plane-wave cutoff is set to $R_{mt}\times K_{max}= 7$ with $G_{max}= 24$. $\text{A} \, 10\times 10\times 9$*k*-space grid with 102 points within the irreducible wedge of the Brillouin zone is found to be well converged. Along with this, full relaxation of the atomic positions by minimizing the atomic forces up to 2 mRy/a.u is taken into account in each case. Self-consistency is assumed for a total energy convergence of less than $10^{-5} \, \text{Ry}$.

## Supplementary information

Supplementary file
