# Supplementary material for: Robust half-metallicity and magnetic phase transition in Sr2CrReO6 via strain engineering
Source: Sci Rep. 2020 Aug 13;10:13778. doi: 10.1038/s41598-020-70768-7 (PMC7426967; doi:10.1038/s41598-020-70768-7)
Supplement: Supplementary file 1 — Supplementary file [file 41598_2020_70768_MOESM1_ESM.pdf]

Supporting Information

Robust Half-Metallicity and Magnetic Phase Transition in Sr_2CrReO_6 via Strain Engineering

Qurat-Ul-Ain, Shahnila Naseem, and Safdar Nazir*
Department of Physics, University of Sargodha, Sargodha Campus, 40100 Sargodha, Pakistan.

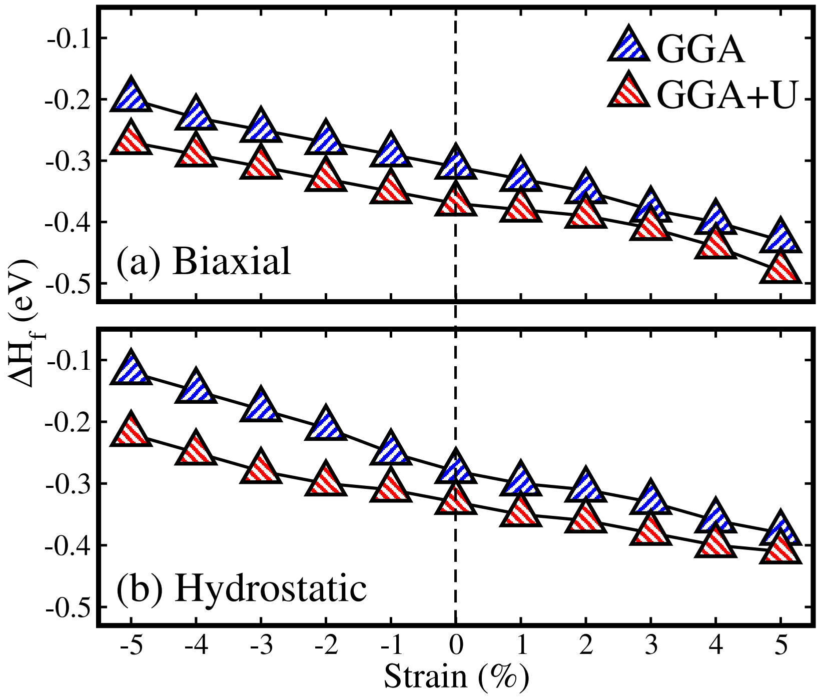

FIG. 1S: Calculated GGA/GGA+U enthalpies of formation (ΔH_f) of Sr_2CrReO_6 DPO in a FiM spin-ordering as a function of (a) biaxial strains along the [110]-direction and (b) hydrostatic compressive strains along the [111]-direction, ranging from -5% to $+5\%$.

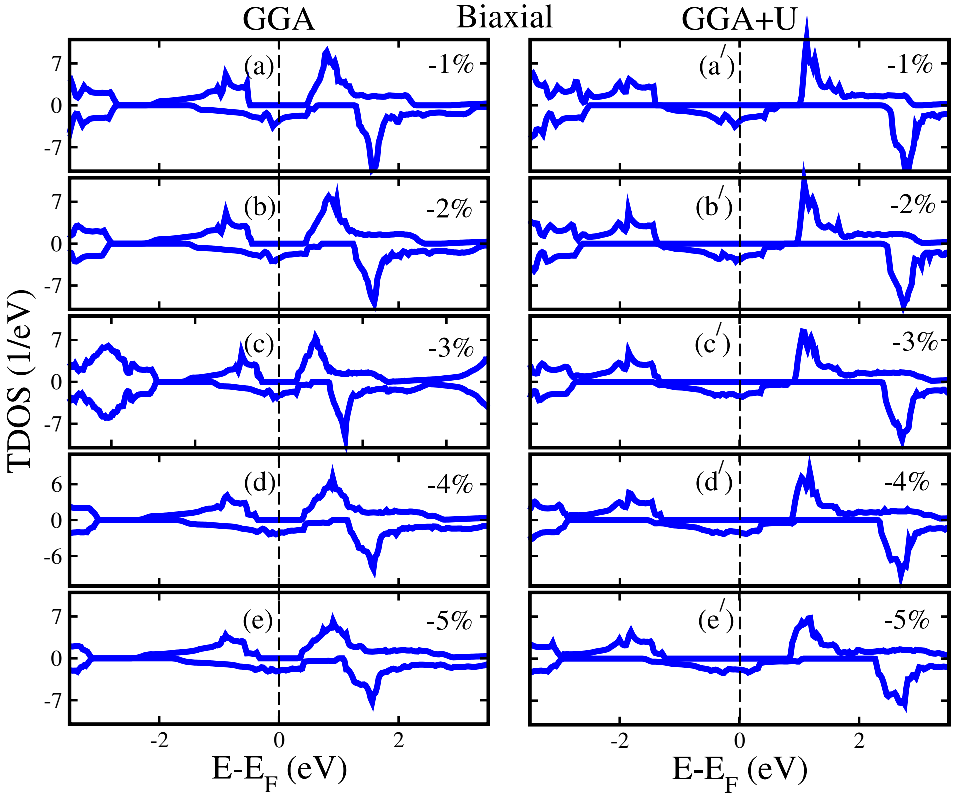

FIG. 2S: Calculated GGA/GGA+U spin-polarized total density of states (TDOS) of Sr_2CrReO_6 for (a/a′) −1%, (b/b′) −2%, (c/c′) −3%, (d/d′) −4%, and (e/e′) −5% biaxial compressive strains along the [110]-direction (in the ab-plane).

---

* Electronic address: safdar.nazir@uos.edu.pk, Tel: +92-334-971-9060

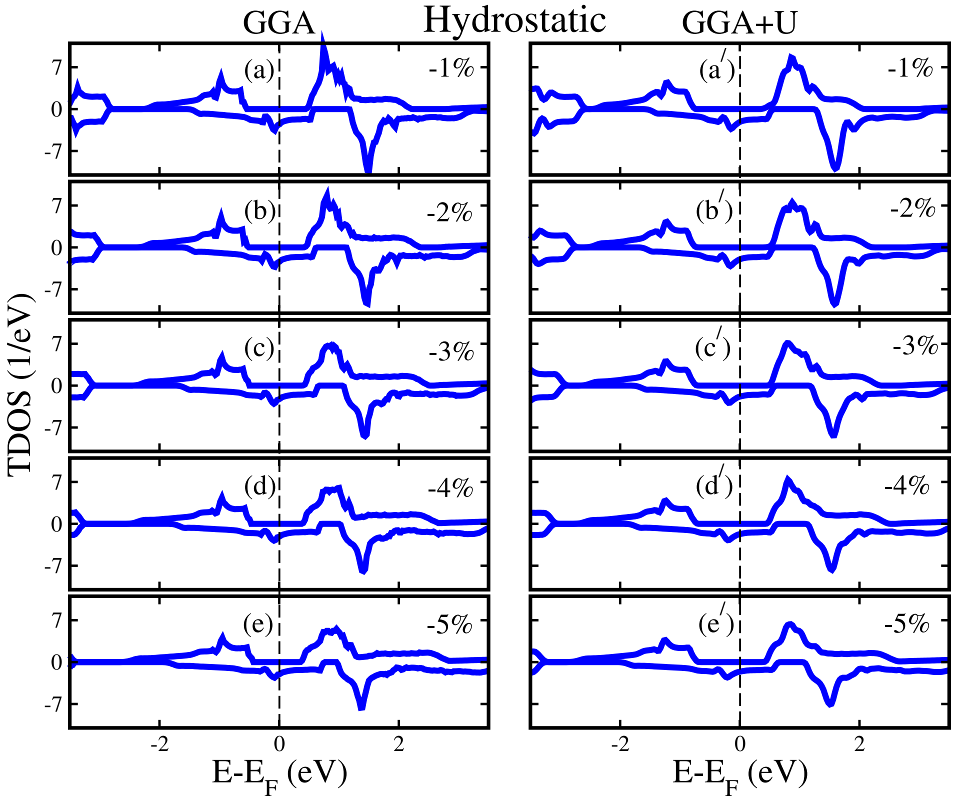

FIG. 3S: Calculated GGA/GGA+U spin-polarized total density of states (TDOS) of Sr_2CrReO_6 for (a/a′) −1%, (b/b′) −2%, (c/c′) −3%, (d/d′) −4%, and (e/e′) −5% hydrostatic compressive strains along the [111]-direction.

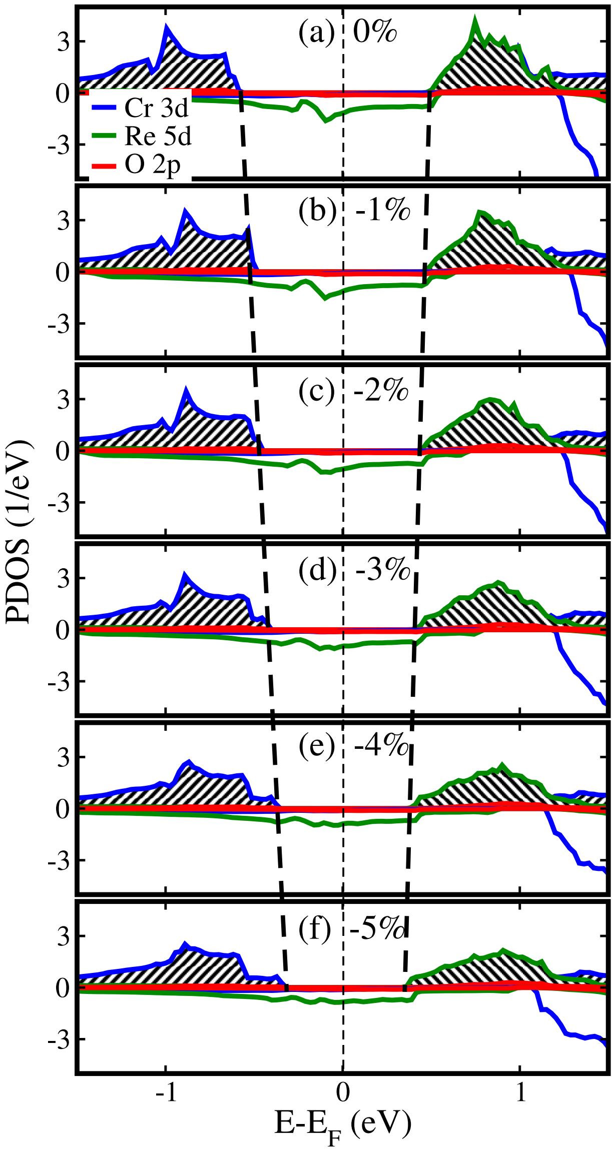

FIG. 4S: Calculated GGA spin-polarized Cr 3d, Re 5d, and O 2p partial density of states (PDOS) of Sr_2CrReO_6 for (a) unstrained (0%), (b) −1%, (c) −2%, (d) −3%, (e) −4%, and (f) −5% biaxial compressive strains along the [110]-direction (in the ab-plane).

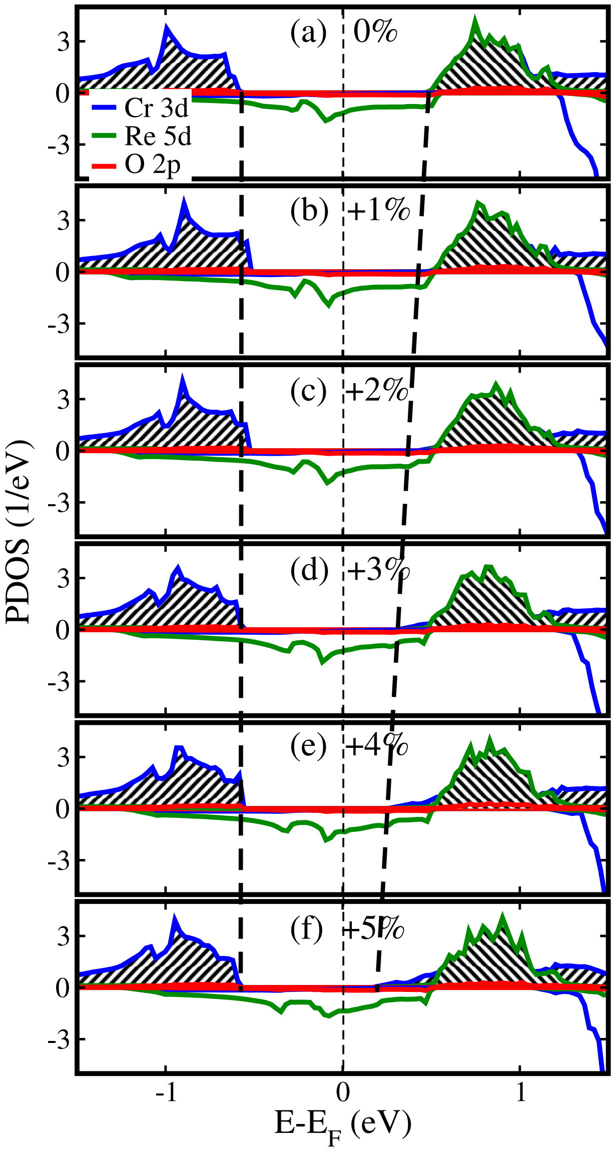

FIG. 5S: Calculated GGA spin-polarized Cr 3*d*, Re 5*d*, and O 2*p* partial density of states (PDOS) of Sr_2CrReO_6 for (a) unstrained (0%), (b) +1%, (c) +2%, (d) +3%, (e) +4%, and (f) +5% biaxial tensile strains along the [110]-direction (in the *ab*-plane).
